# Enhanced Polariton Interactions in Suspended WS_2_ Monolayer Microcavity

**DOI:** 10.1002/adma.202418612

**Published:** 2025-05-02

**Authors:** Laura Polimeno, Francesco Todisco, Rosanna Mastria, Milena De Giorgi, Antonio Fieramosca, Marco Pugliese, Dario Ballarini, Anna Grudinina, Nina Voronova, Daniele Sanvitto

**Affiliations:** ^1^ CNR Nanotec Institute of Nanotechnology via Monteroni 73100 Lecce Italy; ^2^ National Research Nuclear University MEPhI (Moscow Engineering Physics Institute) Kashirskoe shosse 31 115409 Moscow Russia; ^3^ Russian Quantum Center Skolkovo IC, Bolshoy boulevard 30 bld. 1 121205 Moscow Russia

**Keywords:** 2D materials, polariton, suspended monolayer, TMDs

## Abstract

Transition‐metal dichalcogenides monolayers exhibit strong exciton resonances that enable intense light‐matter interactions. The sensitivity of these materials to the surrounding environment and their interactions with the substrate result in the enhancement of excitonic losses through scattering, dissociation and defects formation, hindering their full potential for the excitation of optical nonlinearities in exciton‐polariton platforms. The use of suspended monolayers holds the potential to completely eliminate substrate‐induced losses, offering unique advantages for the exploitation of intrinsic electronic, mechanical, and optical properties of 2D materials‐based polaritonic systems, without any influence of proximity effects. In this work, we report a novel fabrication approach enabling the realization of a planar microcavity filled with a suspended tungsten disulfide (WS_2_) monolayer in its center. We experimentally demonstrate a 2‐fold enhancement of the strong coupling at room temperature, due to the larger exciton binding energy and reduced overall losses as compared to similar systems based on dielectric‐filled microcavities. As a result, spin‐dependent polaritonic interactions are significantly amplified, leading to achievement of a record exciton interaction constant approaching the theoretically predicted value. This approach holds promises for pushing 2D materials‐based polaritonic systems to their intrinsic limits, paving the way for the realization of novel polaritonic devices with superior performance.

## Graphical abstract

Monolayers of transition‐metal dichalcogenides exhibit strong exciton resonances for light‐matter interactions at RT, though substrate effects limit performance. This work presents a planar microcavity with a suspended WS_2_ monolayer, eliminating substrate‐induced losses. The system shows enhancement of strong coupling and preserves the spin‐dependent polaritonic interactions, achieving a record exciton interaction constant close to theoretical predictions. This approach reveals the intrinsic optical and spintronic properties of 2D materials, paving the way for advanced polaritonic.

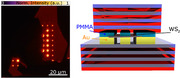

## 1 Introduction

Exciton polaritons are hybrid quasi‐particles arising from the strong coupling between photons and excitons in semiconductors,^[^ ^1^ ^]^ and represent a promising frontier for the development of next‐generation optoelectronic and quantum technologies, working at room temperature (RT).^[^ ^2^, ^3^ ^]^ Among all the unique properties of these hybrid light‐matter states, their strong nonlinear character represents one of the most interesting features, since it opens the way to the observation of many fascinating effects, including superfluidity,^[^ ^4^, ^5^ ^]^ Bose‐Einstein condensation^[^ ^6^ ^]^ and long‐range energy transport.^[^ ^7^ ^]^

Recently, atomically thin transition‐metal dichalcogenides (TMDs) have captured considerable interest from different solid state research communities, including photonics and polaritonics, due to their distinctive optical and structural characteristics.^[^ ^8^, ^9^ ^]^ In fact, TMD monolayers exhibit strong quantum confinement and reduced dielectric screening, leading to large excitonic binding energies (0.6 – 1 eV) that make excitonic resonances robust even at RT.^[^ ^10^, ^11^, ^12^ ^]^ The atomic thickness of these materials and their defined in‐plane oriented dipole, moreover, makes them an ideal active material for probing strong light‐matter interactions in different platforms, ranging from planar microcavities^[^ ^13^, ^14^, ^15^ ^]^ to waveguides,^[^ ^16^ ^]^ metasurfaces,^[^ ^17^ ^]^ Bloch surface waves^[^ ^18^ ^]^ and plasmonic and dielectric nanostructures.^[^ ^19^, ^20^ ^]^.

The principal constraint in employing TMD monolayers as active media in exciton polariton platform is their high interaction with substrates, interfaces, and overall surrounding environment, all contributing to a reduction of the oscillator strength and quenching of the photo‐emission^[^ ^21^, ^22^, ^23^ ^]^. In fact, upon depositing a TMD monolayer onto a substrate, interface effects induce the formation of defect‐mediated localized states, doping effects or uncontrollable strain,^[^ ^24^, ^25^ ^]^ introducing non‐radiative losses channels and localized exciton traps.

Until now, different fabrication techniques have been employed to minimize the effects of interactions with the substrate, such as encapsulation in multilayer hexagonal boron nitride (hBN),^[^ ^26^, ^27^ ^]^ the use of self‐assembled monolayers on various substrates^[^ ^28^ ^]^ and passivation treatments.^[^ ^29^ ^]^ All these processes, however, rely on the inclusion of additional materials to induce surface modification of the dielectric environment surrounding TMD monolayers, thus suffering from spatial inhomogeneities and introducing additional spacing around the monolayer, that can locally screen near‐field interactions and add proximity effects. In this context, a promising alternative approach is the complete suspension of the monolayer as a free‐standing membrane, effectively eliminating substrate‐induced effects.^[^ ^30^, ^31^, ^32^ ^]^ and leading to increased oscillator strength and exciton binding energy.^[^ ^33^, ^34^ ^]^ This, in turn, provides an optimal platform for investigating excitonic properties in the strong coupling regime in microcavities.

In this study, we report a reliable fabrication process for embedding a suspended tungsten disulfide (WS_2_) monolayer in the middle of a planar microcavity, allowing us to explore the ultimate limits of TMD‐based microcavity polaritons. In such a configuration, we theoretically simulate and experimentally demonstrate strong light‐matter interactions of the suspended monolayer, showing higher oscillator strength as compared to dielectric‐filled microcavities and enhanced optical nonlinearities and polariton‐exciton interactions. By completely suppressing substrate‐induced effects, the excitonic losses are minimized and we demonstrate a record Rabi splitting of 56 meV for a planar microcavity based on a WS_2_ monolayer, achieving twice the Rabi splitting of a microcavity in which the TMD is monolithically embedded rather than suspended. Moreover, using both conservative and direct experimental methods to determine the polariton density, the nonlinear interactions in the suspended monolayer‐based cavity are significantly stronger than those reported for WS_2_ monolayers at RT, demonstrating not only the maximization of the intrinsic oscillator strength of these materials but also the preservation of spin‐dependent interactions.

Our results mark an important first step toward the realization and fabrication of all‐optical polariton platforms that fully leverage the optical properties of TMD monolayers, overcoming all substrate‐induced limitations, including inhomogeneities and dielectric effects. Additionally, we emphasize that the suspended region can be easily prepared with specific geometrical patterns, paving the way for the straightforward realization of various types of suspended structures in structured artificial potentials.

## 2 Results and Discussion

To demonstrate the improvement and enhanced performance of our suspended system, we selected WS_2_ from the range of TMD monolayers because in the encapsulated configuration it has been shown to exhibit weaker exciton interaction (g_*exc*_ ≈ 0.2 µeV µm^2^ at RT).^[^ ^14^, ^15^, ^35^, ^36^ ^]^

First, to investigate the optical properties of suspended WS_2_ monolayer membranes, we etched 300 nm deep holes onto a commercial Si/SiO_2_ substrate, with diameters ranging from 1 to 5 µm (see Section S1, Supporting Information for further details). A WS_2_ monolayer is then mechanically exfoliated and transferred onto the patterned area via the dry transfer method,^[^ ^37^ ^]^ followed by a vacuum hard baking on a hot plate to improve adhesion (3 h at 200° C).

**Figure 1:**
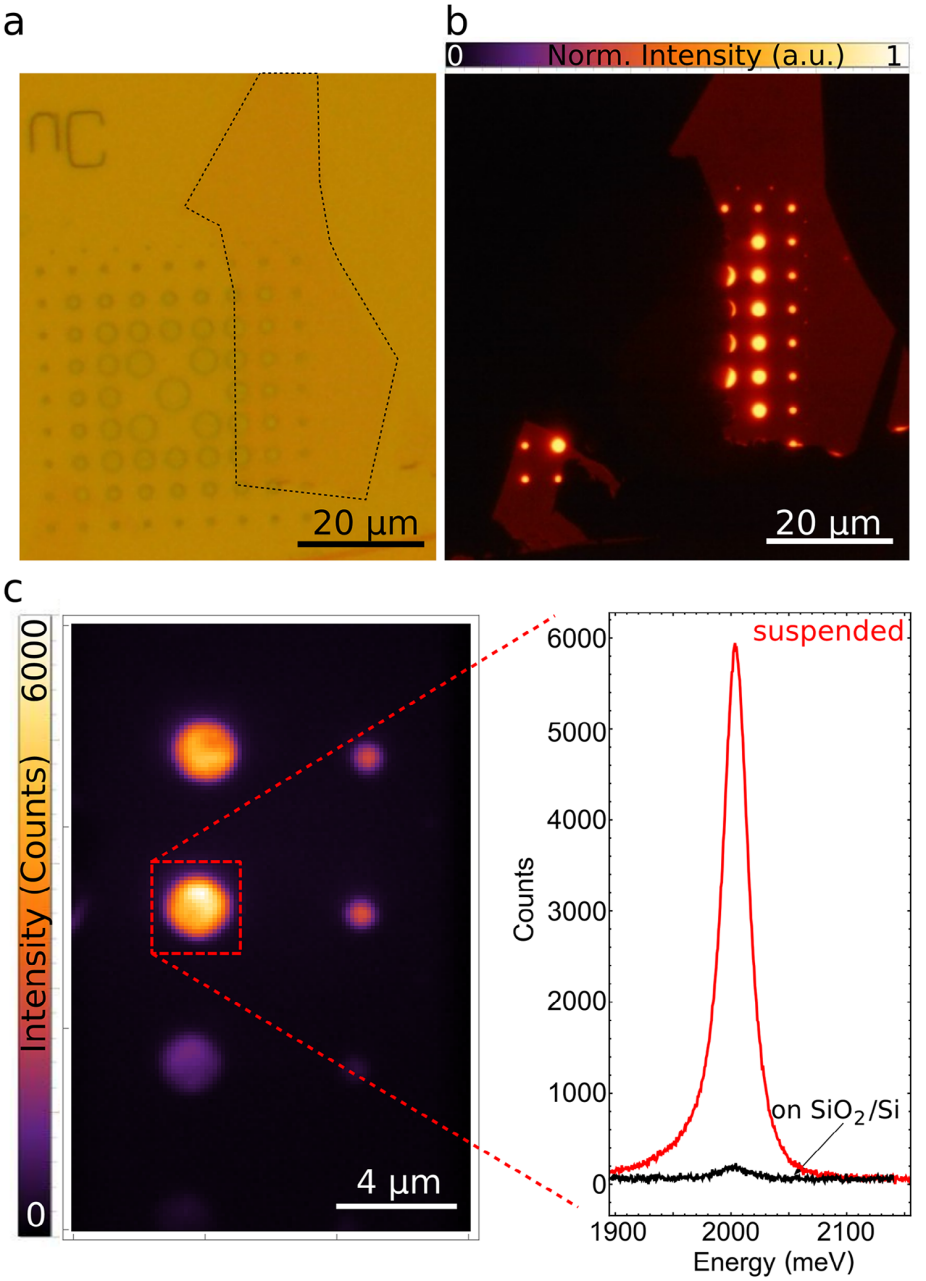
Real space bright‐field reflection (a) and photoluminescence (b) image of a suspended WS_2_ monolayer at RT. The black dashed line in (a) indicates the contour of the monolayer flake transferred on the holes array. The dimension of the holes ranges from 1 µm to 5 µm. c) Zoom of the monolayer photoluminescence on a 2 µm hole. The monolayer is off‐resonantly pumped with a CW laser at ≈ 2541 meV, with a 100 µm spot. d) Comparison of the monolayer photoluminescence spectra recorded from suspended (red) and SiO_2_/Si substrate (black) regions.

**Figure** **1**a shows the bright‐field reflection image of the monolayer (black dashed line) deposited on the holes array. The corresponding photoluminescence (PL) image in true colors, recorded with a color CCD camera on an optical microscope equipped with a blue LED light source and a colored filter, is shown in Figure 1b, perfectly following the holes pattern and demonstrating the enhancement of the PL in the suspended area.

To quantitatively evaluate the PL enhancement, the sample is off‐resonantly excited with a continuous wave laser (energy peak at 2541 meV) with a spot size of ≈100 µm, exciting both the free‐standing and substrate‐attached regions, as shown in Figure 1c. Under the same pumping conditions (excitation density ≈10 nWµm^−2^), the PL intensity from the suspended WS_2_ is ten times stronger than that from the attached one. Additionally, the PL lineshape of the suspended WS_2_ is narrower due to a decrease in the trion contribution to the PL, attributed to a reduction in unintentional doping and strain (Figures S1 and S2, Supporting Information). This marked difference in photoemission efficiency provides clear evidence that excitonic loss channels are largely suppressed in the suspended monolayer, which remains fully isolated from its environment.^[^ ^30^, ^31^ ^]^ As a consequence, free‐standing monolayer membranes appear to be ideal active platforms for improving strong light‐matter interactions in planar microcavities.

**Figure 2:**
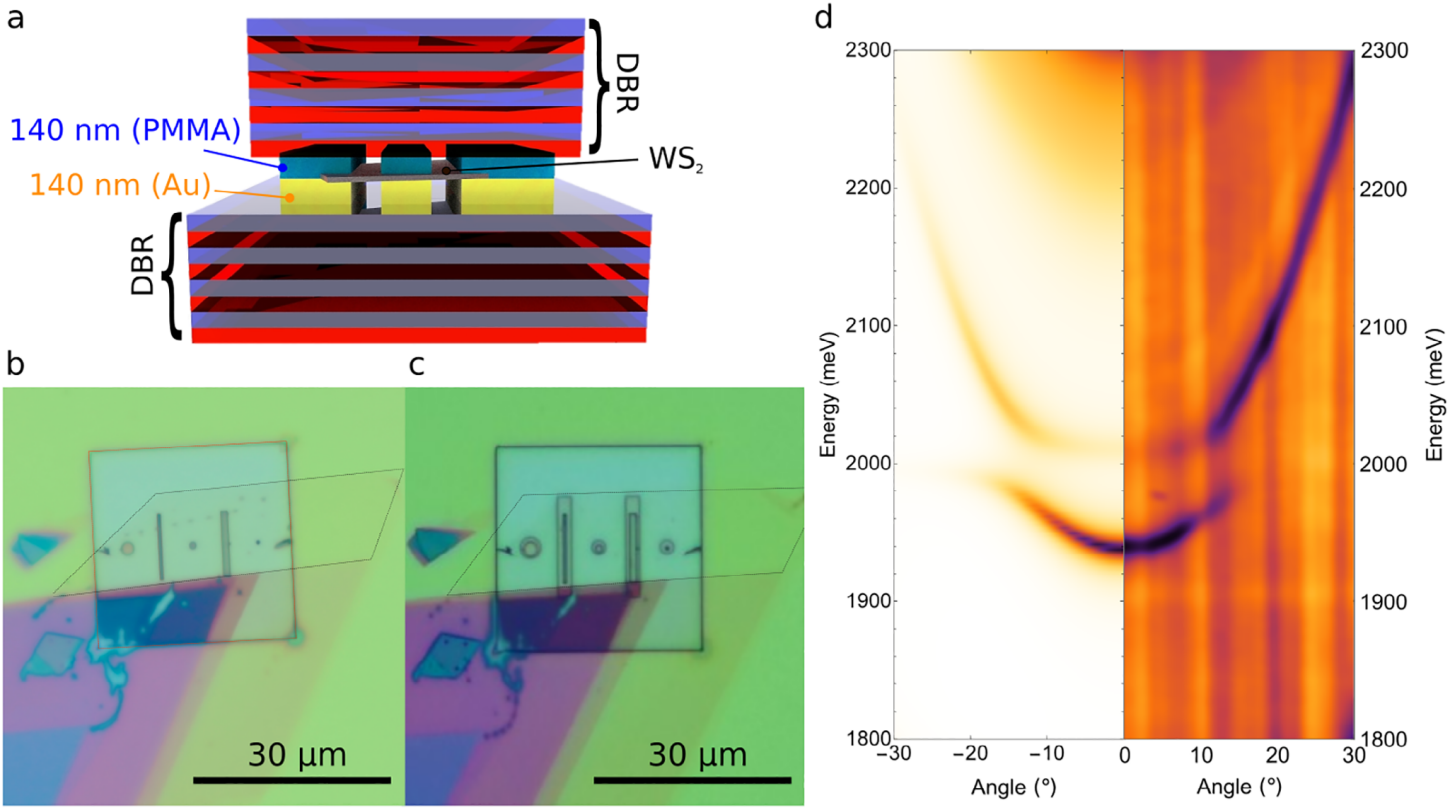
a) Sketch of the planar microcavity. The 140 nm‐thick gold island is fabricated on top of a DBR made of four pairs of SiO_2_/TiO_2_ layers. The WS_2_ monolayer is suspended inside the planar microcavity at the position where the confined electromagnetic field is maximum. b) Real space image of the sample after mechanical transfer. The monolayer flake is transferred onto two empty stripes of 1 × 10 µm and 0.5 × 10 µm, etched into the gold island. c) Real space image of the sample after writing the top PMMA using EBL. The top stripes are larger than the bottom ones to facilitate the alignment of the suspended region. d) Comparison between the theoretical simulation (left‐hand side) and the experimental reflectivity (right‐hand side) in Fourier space of the 1 × 10 µm stripe. The white light (spot dimension ≈1 µm) is focused on the suspended region.

The design of our planar microcavity is illustrated in **Figure** **2**a (all fabrication steps are described in Section S2, Supporting Information), where the top and bottom mirrors consist of two Distributed Bragg Reflectors (DBRs), each made of four pairs of SiO_2_/TiO_2_ layers. In such a system, the most crucial parameter for maximizing light‐matter interactions is the position of the free‐standing membrane, that needs to be placed at the maximum of the confined electromagnetic field. In particular, by using high refractive index terminated DBRs, we found the optimum air‐filled exciton‐resonant microcavity total thickness to be 280 nm, corresponding to a λ/2 cavity.

In order to manufacture such a platform, a 30 µm × 30 µm gold pad with two rectangular holes of 1 × 10 µm and 0.5 × 10 µm is fabricated on the surface of a DBR by electron beam lithography (EBL) followed by 140 nm metal evaporation (3 nm chromium followed by 137 nm gold) and lift‐off (see Experimental Section for details). A WS_2_ monolayer is then mechanically transferred on the structured pad (as highlighted by a black dashed line in Figure 2b), followed by vacuum hard baking to improve adhesion (Figure S4, Supporting Information). Then, a 140‐nm thick Polymethyl‐methacrylate (PMMA) top layer is spin coated on the sample and two stripes are defined by electron beam lithography on top of the free‐standing monolayer regions. In order to maximize the suspended area and minimize alignment errors, the top stripes are intentionally defined larger than the bottom ones, as shown in Figure 2c. The microcavity is finally closed by mechanical transfer of the top DBR onto the structured area, as already reported in previous works.^[^ ^13^, ^14^, ^38^, ^39^ ^]^

The theoretical simulation of the Fourier space reflectivity dispersion of the designed microcavity is shown in left panel of Figure 2d. The energy of the optical mode is tuned to the WS_2_ excitonic resonance (≈2000 meV), but strong coupling is achieved only in the suspended region, where the maximum of the electromagnetic field coincides with the spatial position of the monolayer (see Section S3, Supporting Information). The right half of Figure 2d shows the measured angular dispersion of the reflectivity for the 1 × 10 µm stripe. The long dimension of the stripe is aligned with the entrance of the slit, and a focused white light (spot size reduced to 1 µm) is used to select only the signal coming from the suspended region. The angular dispersion clearly shows the bending of the optical mode at the excitonic resonance (Exc ≈ 2000 meV) at angle of θ ≈ 15°, in good agreement with the simulation. As theoretically predicted, the planar microcavity is perfectly tuned with the WS_2_ exciton in the entire suspended region, while only the optical mode is measurable in the substrate‐attached area (Figure S5 in Section S3, Supporting Information).

**Figure 3:**
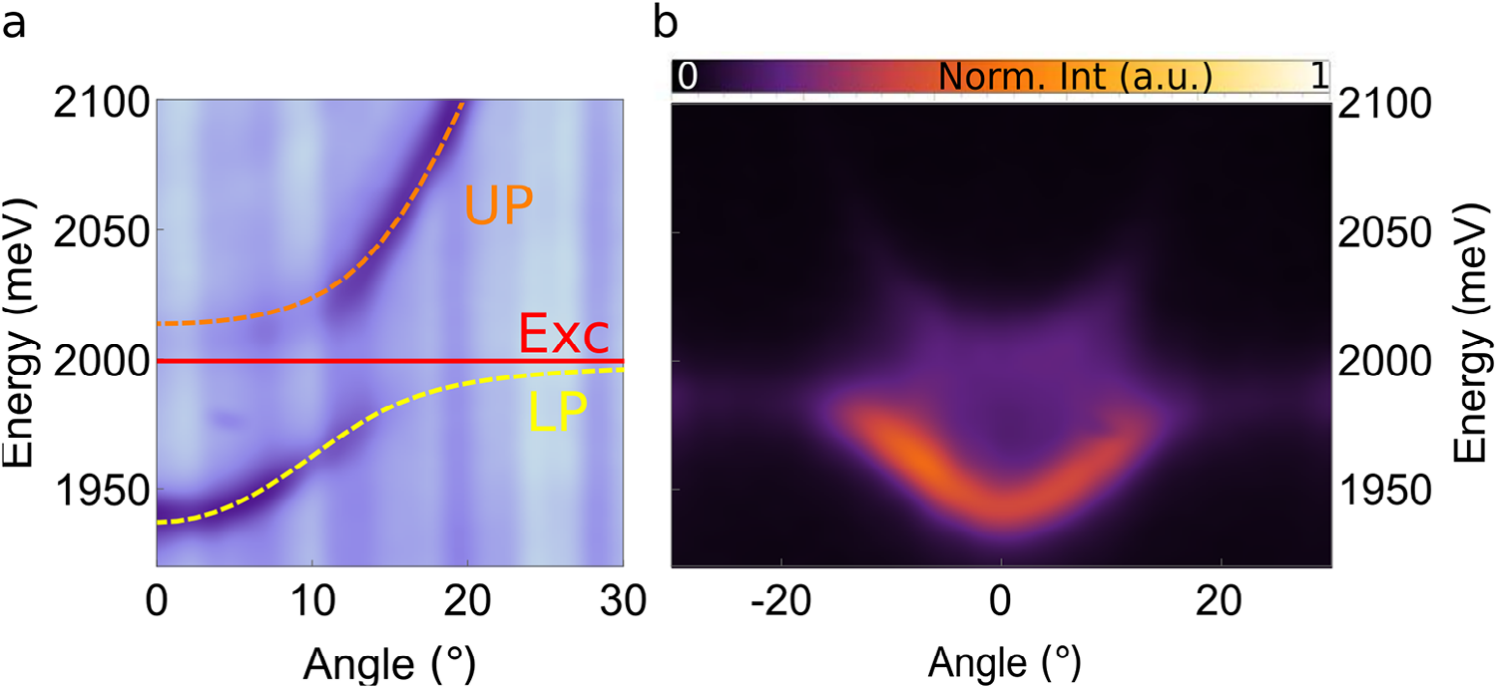
a) Fitting of the experimental reflectivity with a two coupled oscillators model. The strong coupling between the excitonic resonance (Exc) of the suspended monolayer (continuous red line) and the optical mode confined in the cavity leads to the formation of the upper polariton (orange dashed line) and lower polariton (yellow dashed line) branches, with a Rabi splitting of 56 meV. b) Measured angular dispersion photoluminescence of WS_2_ suspended monolayer in the planar microcavity.

The anticrossing of the optical mode with the monolayer exciton is fitted using a two‐coupled oscillators model (see Experimental Section for further information), as shown **Figure** **3**a. The dashed yellow and orange lines represent the fitting of the lower and upper polariton branches, respectively, extracting a Rabi splitting of Ω_*R*_ ≈56 meV. The Ω_*R*_ found for the suspended monolayer in the strong coupling regime is twice that of a typical planar microcavity where the monolayer is embedded within two dielectric‐filled DBRs,^[^ ^40^, ^41^, ^42^ ^]^ demonstrating a strong enhancement of the exciton oscillator strength with respect to the typical microcavity, in which the optical confined mode is comparable. By off‐resonantly pumping the system with a CW laser (peak energy at 2541 meV), we observe photoemission from the lower polariton branch in the angle‐resolved dispersion (Figure 3b), highlighting the enhanced PL efficiency. The air layers above and below the monolayer act as a protective buffer, shielding the exciton from uncontrolled doping, strain, and defect formation–common issues in conventional SiO_2_‐ or PMMA‐filled microcavities. In our suspended system, the monolayer is fully isolated, maximizing the strong coupling with the optical mode confined in the microcavity.

To further investigate the effects of the reduction of substrate scattering and losses on the polaritons optical properties, we measured the polarization‐dependent polariton‐polariton interactions in our suspended platform. The underlying mechanism governing nonlinear interactions in TMD monolayer‐based exciton‐polaritons is highly debated, with different results and interpretations reported in literature to date.^[^ ^43^, ^44^, ^45^ ^]^ However, it can be expected that reduced substrate scattering can improve the polarization coherence of polaritons, leading to stronger polarization‐dependent nonlinearities when resonantly pumping with circularly polarized light. In fact, using a linearly polarized excitation—which is a coherent mix of two oppositely polarized circular polarizations—only half of the excited polaritons will have the same spin, which effectively reduces the interaction energy by approximately a factor of two.

**Figure 4:**
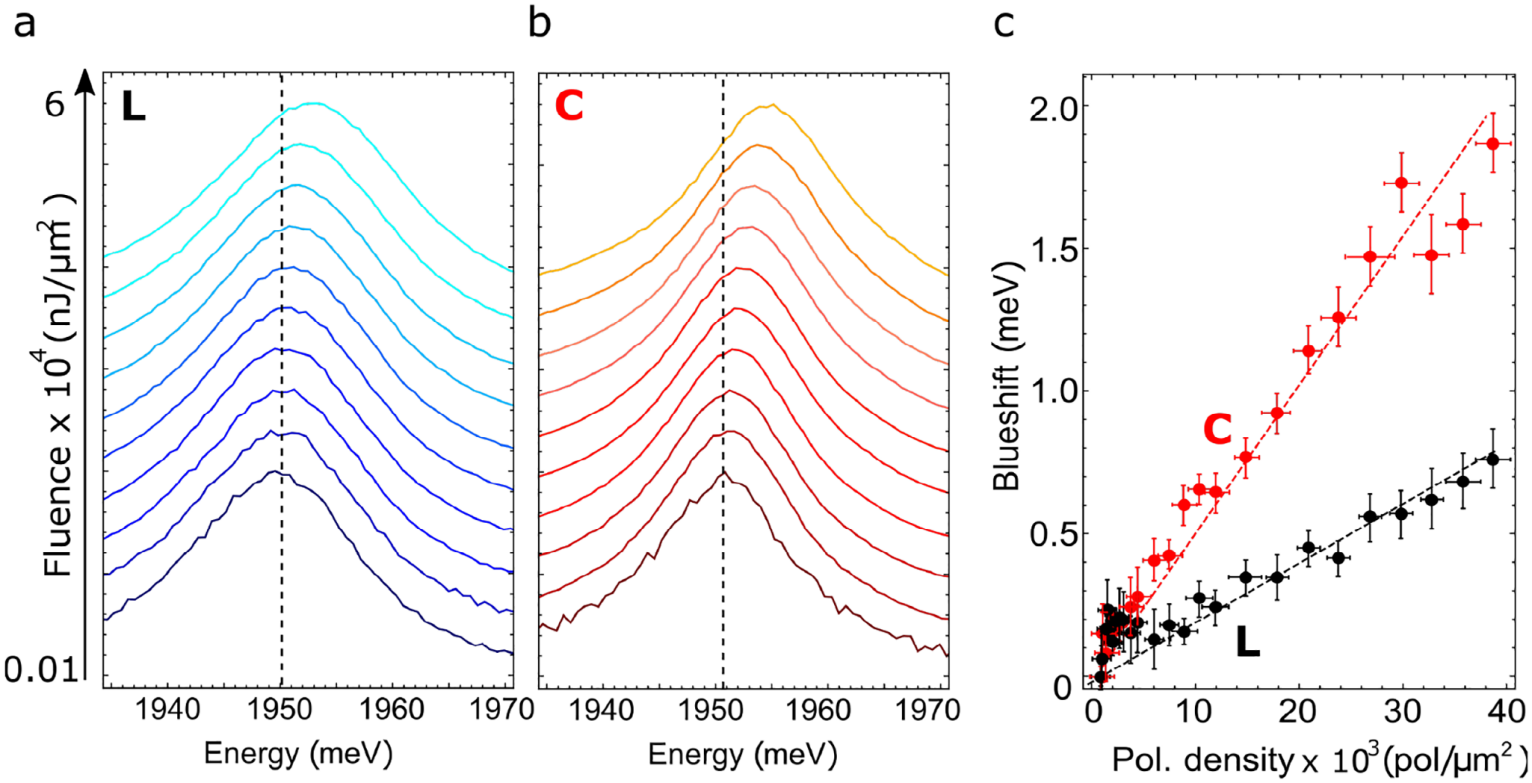
a, b) Transmitted spectra of the pulsed laser on polariton mode at the angle ≈ 0°, corresponding to different resonant pumping fluences for linearly‐ (a) and circularly‐ (b) polarized excitation laser. c) Energy blueshift of the polariton modes at θ ≈ 0° as a function of the polariton density inside the cavity, in the case of linearly‐ (L, black dots) and circularly‐ (C, red dots) polarized excitation. The measurement error is greater at low polariton density compared to higher polariton densities. The dashed lines are linear fits to the experimental data, with the slope yielding the polariton interaction constant, *g*_pol, lin_ = 0.017~µeV· µm^2^ for the linear case and *g*_pol, circ_ = 0.046∼µeV· µm^2^ for the circular one.

Interestingly, deviations from this standard scenario can occur when the nonlinear response of polaritons is influenced by alternative microscopic mechanisms, such as dipolar interactions, screening of the exciton binding energy, or indirect exchange interactions mediated by biexciton states. This is particularly relevant in the case of TMD polaritons, where previous research suggests that interactions involving trions or polarons may play a crucial role.^[^ ^15^ ^]^ To investigate this effect in our real 2D geometry, the lower polariton branch on the suspended region was resonantly pumped at θ ≈ 0° with a resonant fs‐pulsed laser in transmission configuration, at different incident fluences, with the incident laser linear and circular polarization. The corresponding fluence‐dependent transmission spectra are shown in **Figure** **4**a,b, respectively. In both cases, as the fluence increases, the lower polariton branch continuously shifts toward higher energies (blueshifts) due to the the combined effect of the increasing polariton interactions and the so‐called exciton saturation (often referred to phase‐space filling^[^ ^46^, ^47^ ^]^).

As the fluence increases, the larger full width half maximum (FWHM) observed for linear polarization compared to circular polarization is attributed to the differing intensity of excitation‐induced dephasing effect (see Section S4, Supporting Information). The measured blueshift thus allows the estimation of the lower‐polariton nonlinearity, if an accurate knowledge of the polariton density can be acquired. To experimentally evaluate this density in our system, we directly measured the transmitted laser fluence *F* passing through the microcavity defined by an empty (without suspended TMD monolayer) stripe with a rectangular shape of 1 µm × 5 µm. Consequently, the upper bound for the polariton density can be defined as *F*/*E*_*ph*_, where the energy of the resonant pumping laser is *E*_*ph*_ ≈ 1950 meV. It is important to note that this model assumes that all photons entering the system are converted into polaritons, providing an upper limit for the maximum polariton density as function of the fluence.

Defining the blueshift of the polariton dispersion as Δ*E*_*pol*_ = *g*_*pol*_ · *n*_*pol*_, where *g*_*pol*_ is the effective strength of the polariton nonlinearity, by linear fitting of the data reported in Figure 4a,b as a function of the experimental polariton density (Figure 4c) we estimate at RT *g*_*pol*, *lin*_ ≈ 0.017∼µeV µm^2^ and *g*_*pol*, *circ*_ ≈ 0.046∼µeV µm^2^ for the linearly and circularly polarized excitation, respectively. The energy blueshift achieved with circularly polarized excitation is thus substantially higher than that observed when exciting with a linearly polarized laser. This implies that the energy blueshift is substantially larger when all polaritons have the same spin. This result aligns with the typical behavior of polariton–polariton interactions in standard semiconductors at cryogenic temperatures, characterized by a strong repulsive interaction for same‐spin polaritons and a weaker interaction for opposite‐spin polaritons.^[^ ^48^, ^49^, ^50^ ^]^

Note that spin‐dependent interactions are completely suppressed in a typical dielectric‐filled WS_2_ microcavity, as shown in Figure S6 in Section S4 (Supporting Information). It has been anticipated that in this case the main mechanism could be phonon‐mediated interactions induced by the presence of the substrate, which are spin‐independent, as recently experimentally and theoretically demonstrated by Zhao et al.^[^ ^51^ ^]^ In a monolithic cavity, the effective nonlinear response is primarily governed by spin‐independent polaron interactions, resulting in exciton dephasing and the suppression of the spin‐dependent interactions characteristic of polaritonic systems. However, in our suspended system, these interactions are entirely suppressed due to reduced phonon scattering. As a result, our platform retains spin‐dependent interactions, where either phase space‐filling or exchange interactions play a dominant role. This comparison demonstrates that eliminating uncontrollable substrate interactions is essential for preserving spin‐dependent interactions, significantly reducing phonon scattering, and thereby leading to pure polariton‐like nonlinearities.^[^ ^51^ ^]^

Generally, in the mean field approximation the lower‐polariton blueshift^[^ ^52^ ^]^ Δ*E*_*pol*_ = *g*_*pol*_ · *n*_*pol*_ contains two contributions since *g*_*pol*_ = *g*_*exc*_|*X*|^4^ + 2*g*_*sat*_|*C*||*X*|^3^, where *g*_*exc*_ and *g*_*sat*_ are the exciton‐exciton interaction and exciton saturation constants (see Section 5 of the Supporting Information), *X* and *C* are the exciton and photon Hopfield coefficients, respectively, here defined for zero wavevector. With the detuning of the pumped mode δ ≈ −50 meV and the observed Rabi splitting Ω ≈ 56 meV, the excitonic fraction of the lower polariton branch at θ = 0° is |*X*|^2^ = 0.167. Accounting for both contributions, our theory provides the result $g_{pol,circ}^{\mathrm{theor}}=0.04629\;\mu \mathrm{eV}$ µm^2^ which is in perfect correspondence to a measured number. This allows us to extract the values of both *g*_*exc*_ = 1.11338~µeV µm^2^ and *g*_*sat*_ = 0.12229~µeV µm^2^ for our experimental setting. We note that while *g*_*sat*_ is an order of magnitude smaller, its contribution to the blueshift at our large negative detuning is almost 30% (see Section S5, Supporting Information). Thus the *g*_*exc*_ value extracted for the suspended WS_2_ is at least six times higher than the *g*_*exc*_ experimentally extracted as an overestimate (i.e., without accounting for saturation) for both a standard WS_2_ monolayer‐based microcavity (see Section 4 of the Supporting Information)^[^ ^14^, ^36^ ^]^ and also for other polariton platforms based on TMDs.^[^ ^18^, ^35^ ^]^ Furthermore, the measurement of the blueshift slope at linear excitation *g*_*pol*, *lin*_ allows to extract the interaction constant for polariton with opposite spins. The value extracted from the measurement is then α_2_ = 2*g*_*pol*, *lin*_ − *g*_*pol*, *circ*_ = −0.012~µeV µm^2^. Theoretically, a way to estimate the interaction constant for opposite‐spin polaritons was suggested in Ref. [53] and it provides $\alpha_{2}^{\mathrm{theor}}=-0.026\;\mu \mathrm{eV}$ µm^2^ (see Section 5 of Supporting information). We note that our experimental estimation likely overstates the polariton density by neglecting, among other things, also the exciton–exciton annihilation, therefore it corresponds to a conservative evaluation of the interaction strengths.Therefore, our suspended polariton platform at RT greatly enhances polariton interactions.

## 3 Conclusion

Investigating the optical properties of suspended WS_2_ monolayers is essential for optimizing and enhancing exciton‐polariton interactions in TMD‐based microcavity.

In this work, we first demonstrate how to enhance the excitonic emission of WS_2_ by suspending the monolayer as a free‐standing membrane. This isolation resulted in a tenfold increase in PL intensity compared to contacted regions, while maintaining the PL shape.

Then, we develop a novel fabrication approach to introduce a suspended monolayer in a planar microcavity, ensuring that the maximum of the confined electromagnetic field is precisely positioned at the suspended monolayer region.

The experimental and theoretical analysis confirms strong coupling between the suspended WS_2_ exciton and the optical mode, achieving a Rabi splitting of 56 meV. This significantly surpasses the values observed in traditional WS‐based microcavities with comparable optical mode confinement, highlighting how suspension enhances the excitonic properties and strengthens the coupling to light.

Furthermore, our system demonstrated a substantial reduction in excitonic losses and enhanced polariton interactions, as evidenced by the sixfold increase in the exciton interaction constant (*g*_*exc*_) compared to standard WS_2_ monolayer‐based microcavities and exciton‐polariton platform working at RT. Moreover, the spin‐dependent interactions are preserved by eliminating the substrate interaction.

Overall, our suspended polariton platform overcomes the limitations of typical polariton systems by eliminating environmental interactions and enhancing the intrinsic properties of the WS_2_ monolayer.

This improvement underscores the potential of suspended monolayers in strong coupling regime for advanced photonic applications, including topological photonics, photonic crystals, and valleytronics.

## 4 Experimental Section

### Sample Fabrication

The DBR was formed by four pairs of SiO_2_/TiO_2_ layers (with thicknesses of 106 nm/68 nm, respectively), deposited by electron‐beam evaporation (Temescal Supersource), keeping the chamber at 10^−5^ ÷ 10^−6^ mbar throughout the process, with the sample at room temperature and in absence of any oxigen gas flow (deposition rates: 1 Ås^−1^ for SiO_2_, 0.5 Ås^−1^ for TiO_2_). resulting DBR stopband was centered at 2000 meV (620 nm).

The suspended TMD‐based microcavity was defined by a two step litography process. The full details of this procedure is detailed in Section S2 (Supporting Informations). Single‐layer WS_2_ was mechanically exfoliated from bulk crystals (HQ Graphene) with Nitto SPV 224 tape and transferred onto the surface of a PDMS stamp (Gelfilm, retention level x4 from Gel‐Pak®). Single‐layer WS_2_ was transferred by all‐dry deterministic transfer^[^ ^37^ ^]^ on the gold island by using a micromanipulator. The sample was finally annealed in vacuum at 200 °C for 3h.

### Optical Measurements

All measurements reported in this work were performed under ambient conditions at RT.

For photoluminescence measurements (both in real and Fourier space), the WS_2_ monolayer is off‐resonant excited by using a continuous‐wave 488 nm diode laser. The photoluminesce was recorded in reflection configuration, using a 100x objective with NA = 0.5.

The reflectivity measurements of the polariton dispersion were performed using an home‐built microscope, equipped with a 0.5 numerical aperture (NA) objective with 100x magnification. Four lenses were used to project a magnified image of the back focal plane (BFP) onto the slit of an imaging spectrometer with a cooled charge‐coupled device camera.

For nonlinear measurements, a tunable femtosecond laser (with pulse width ≈145 fs, repetition rate 10 kHz) was focused onto the BFP of the 0.55 NA objective at θ ≈ 0. The energy of the laser resonantly pumping the interested polariton mode and the corresponding real space spot size dimensions are ≈1950 meV and ≈ 10 µm^2^, respectively.

### Theoretical Simulation and Fits

Simulations of the reflectivity were instead performed by an open source implementation of the RCWA method.^[^ ^18^, ^54^ ^]^

The experimental data reported in Figure 3a of the main text are fitted with a 2x2 coupled harmonic oscillator Hamiltonian:

(1) $$\begin{array}{c} H_{k}=\left(\begin{array}{cc} E_{ph}\left(\theta \right) & \Omega_{R}/2\\ \Omega_{R}/2 & Exc \end{array}\right) \end{array}$$

where *Exc* is the exciton energy, Ω_*R*_ is the Rabi splitting and *E*_*ph*_(θ) is the energy dispersion of the photonic branch.

## Conflict of Interest

The authors declare no conflict of interest.

## Supporting information

Supporting Information

## Data Availability

The data that support the findings of this study are available from the corresponding author upon reasonable request.
