# Supplementary material for: Enhanced Polariton Interactions in Suspended WS2 Monolayer Microcavity
Source: Adv Mater. 2025 May 2;37(27):2418612. doi: 10.1002/adma.202418612 (PMC12243721; doi:10.1002/adma.202418612)
Supplement: Supplementary file 1 — Supporting Information [file ADMA-37-2418612-s001.pdf]

ADVANCED MATERIALS

Supporting Information

for *Adv. Mater.*, DOI 10.1002/adma.202418612

Enhanced Polariton Interactions in Suspended WS_2 Monolayer Microcavity

Laura Polimeno, Francesco Todisco*, Rosanna Mastria, Milena De Giorgi, Antonio Fieramosca, Marco Pugliese, Dario Ballarini, Anna Grudinina, Nina Voronova and Daniele Sanvitto*

Supporting Information: Enhanced polariton interactions in suspended WS_2 monolayer microcavity

Laura Polimeno[1†*], *Francesco Todisco*[1†*], *Rosanna Mastria*[1], *Milena De Giorgi*[1], *Antonio Fieramosca*[1], *Marco Pugliese*[1], *Dario Ballarini*[1], *Anna Grudinina*[2,3], *Nina Voronova*[2,3], *Daniele Sanvitto*[1]

[1]CNR Nanotec, Institute of Nanotechnology, via Monteroni, 73100, Lecce, Italy.
[2]National Research Nuclear University MEPhI, (Moscow Engineering Physics Institute), Kashirskoe shosse 31, 115409 Moscow, Russia.
[3]Russian Quantum Center, Skolkovo IC, Bolshoy boulevard 30 bld. 1, 121205 Moscow, Russia.

1 Holes pattern

Circular holes or stripes pattern was etched on the surface of commercial silicon dioxide (300 nm thick) on silicon wafers. The substrates were cleaned by sonication in acetone and 2-propanhol, followed by oxygen plasma (200 W, 100 sccm, 5 minutes). The sample was then spin coated with 300 nm thick polymethyl methacrylate (950K PMMA A4, MicroChem) and soft baked at 180 °C for 15 minutes. Electron beam litography was then carried out using a 250 μC beam dose and development in a 2-propanhol water 3:1 mixture at room temperature (30 sec). The holes pattern was finally etched in silicon dioxide by wet etching in a 6:1 buffered oxide etchant for 180 sec (Sigma Aldrich). The

† These authors contributed equally: L. Polimeno, F. Todisco
* e-mail: laura.polimeno@nanotec.cnr.it, francesco.todisco@nanotec.cnr.it

PMMA mask was finally removed by immersion in hot acetone for 10 minutes.

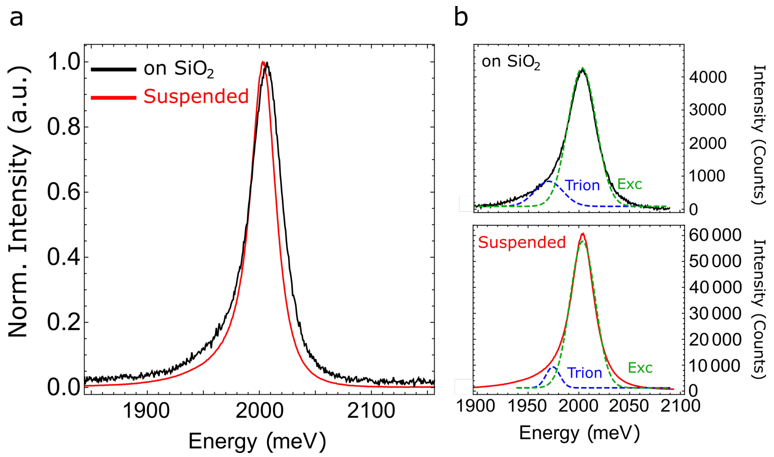

Figure S1: a) Comparison of the normalized PL spectra between the suspended region (red continuous line) and the SiO_2/Si substrate (black continuous line) for analyzing the PL lineshape. b) Fit functions of the experimental data for exciton (green dashed line) and trion (blue dashed line) in both cases.

To demonstrate the impact of the dielectric environment on the WS_2 monolayer exciton, we compare the emission of the suspended monolayer with that of the monolayer encapsulated in hexagonal boron nitride (hBN) (Fig. S2). By modifying the top and bottom environments with a material with high refractive index (~ 2.1), the exciton peak exhibits a redshift compared to the suspended case. Additionally, the emergence of the trion peak is associated with defects and bubbles at the interface between the monolayer and the hBN flake.

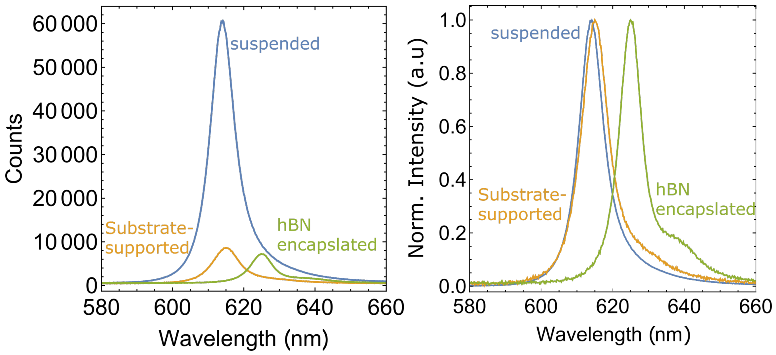

Figure S2: a) Comparison of the PL spectra between the suspended region (blue continuous line), the substrate-supported (orange continuous line) and hBN encapsulated monolayer (green continuous line).

2 Sample fabrication

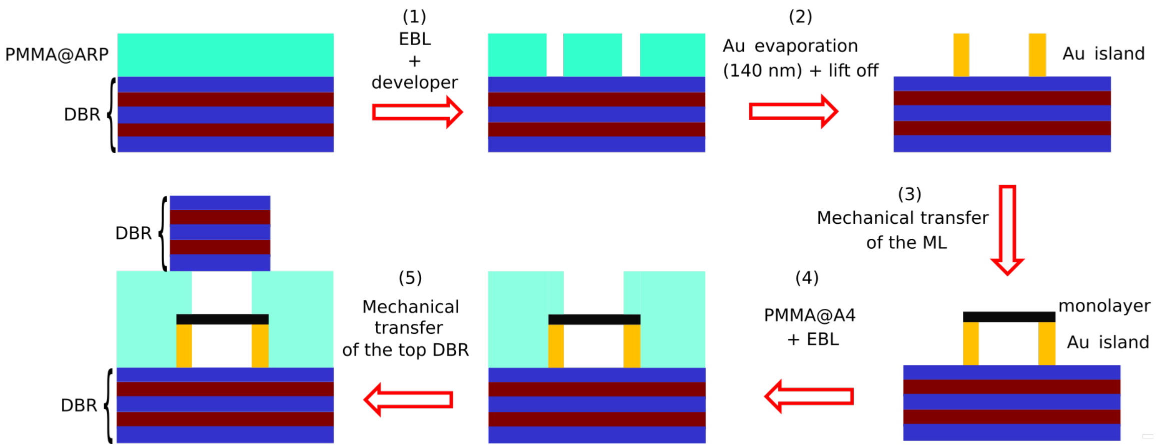

Figure S3: Step by step fabrication process of WS_2 suspended monolayer embedded in a planar microcavity.

Fig. S3 shows, step by step, the fabrication process developed for the realization of the suspended monolayer-based planar microcavity. (1) A 500 nm-thick PMMA ARP671.04 was directly written via Electron Beam Lithography (EBL), imprinting the final design: a 30 μm x 30 μm square with two strips with dimensions of 0.5 μm x 10 μm and 1 μm x 10 μm. (2) A 140 nm-thick gold film is evaporated on the substrate and the lift off process

are made in acetone for 2 hours. (3) The WS_2 monolayer is mechanically transferred on the gold island, suspending the monolayer region below the two strips. The sample is annealed at 200° in vacuum for 3 hours, for improving the adhesion between the gold and the monolayer (Fig. S4). (4) For the top part of the cavity, 140 nm-thick PMMA A4 was

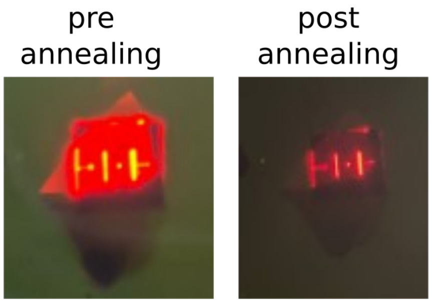

Figure S4: Comparison between the monolayer emission after the transfer and after the annealing.

spun on the top of the monolayer and the EBL writing was aligned with the bottom strips. To optimize the overleap of the suspended area, the top strips were enlarged with respect the first one. The top strips were exposed with a 30kV electron beam, using a positive tone process at low doses; the sample was finally developed with a low-stress/low-dose developer, followed by a surfactant-enhanced deionized water rinsing and N_2 drying. (5) The optical microcavity was closed with the transferred of the DBR, via all-dry transfer.

3 Dispersion in the contacted region

The reflectivity dispersion in the contacted region of the monolayer (Fig. S5, right panel) indicates the presence of the uncoupled optical mode, as theoretically expected (Fig. S5, left panel). In this sample region, the monolayer is in direct contact with the gold island, mostly quenching the exciton emission and removing the air spacer at the bottom of the cavity.

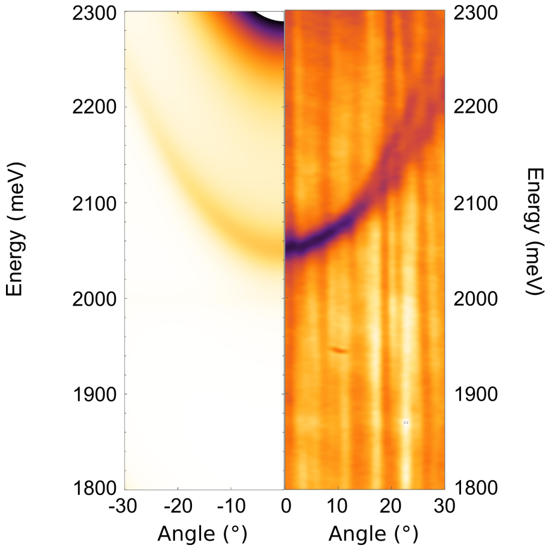

Figure S5: Comparison between the theoretical simulation and the experimental reflectivity in Fourier space of the contacted region.

4 Monolithic microcavity

To directly compare the spin-dependent interactions of the suspended microcavity with the monolithic one, we measure the blueshift of the lower polariton mode of a typical microcavity at RT (Fig. S6a) as a function of the incident power, by varying the polarization of the excitation (Fig. S6b). The Rabi splitting of this cavity is ~ 32 meV, while the detuning is – 62 meV. For this set of data, the polariton-polariton interaction strength is $g_{pol} \sim 0.005\ \mu$eV μm^2 while the excitonic one is $g_{exc} \sim 0.2\ \mu$eV μm^2. By comparing the FWHM for linear and circular polarization at low and high power (Fig. S7), we observe a distinct broadening effect as the pumping power increases - this effect is also observed in the suspended cavity. As the excitation density increases, excitation-induced dephasing (EID) in TMD monolayers causes excitonic linewidth broadening due to many-body interactions, such as exciton-exciton and exciton-carrier scattering[1,2].

In WS_2 monolayers, circularly polarized light excites excitons confined to a single valley

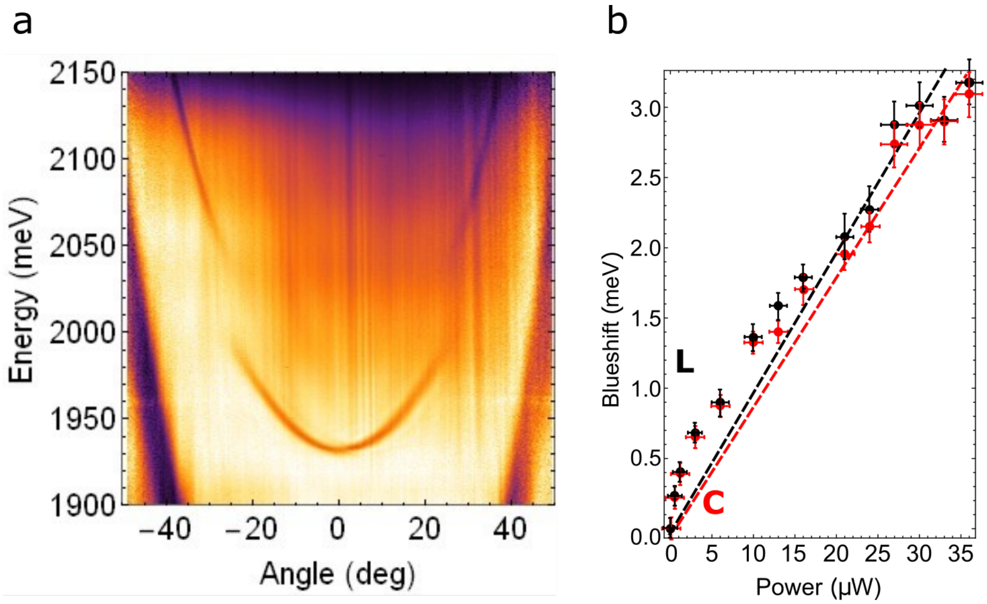

Figure S6: a) Reflectivity in Fourier space of the monolithic cavity.
b) Energy blueshift of the lower polariton as a function of the polariton density inside the cavity, in the case of linearly- (L, black dots) and circularly- (C, red dots) polarized excitation for a monolithic microcavity.

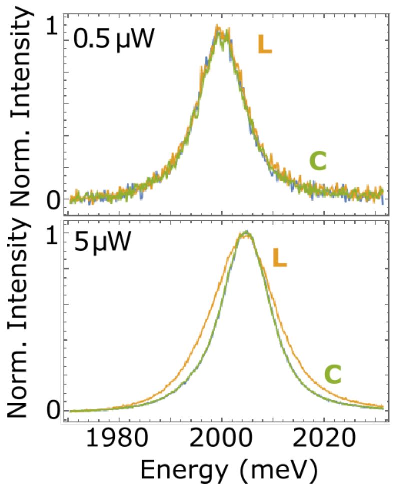

Figure S7: Comparison of the circularly and linearly polarized spectra at low (top panel) and high (bottom panel) power.

(K or K'), limiting exciton-exciton interactions to the same subset of states. In contrast, linearly polarized light, being a superposition of circular polarizations, generates excitons

in both valleys. The simultaneous presence of excitons in opposite valleys allows for more complex exciton-exciton scattering processes, intensifying dephasing under linear polarization and resulting in greater peak broadening compared to circular polarization as the density increases.

5 Exciton and polariton interaction constants

The standard approach to theoretically estimate polariton nonlinearities [3,4] leads to the exciton interaction constant

$$g^0_{exc} = 2\int \frac{d\mathbf{p}}{(2\pi)^2} \int \frac{d\mathbf{q}}{(2\pi)^2} V(\mathbf{q})\chi(\mathbf{p})\chi^*(\mathbf{p})\chi^*(\mathbf{p}-\mathbf{q})\big[\chi(\mathbf{p}) - \chi(\mathbf{p}-\mathbf{q})\big] \tag{S1}$$

and the saturation constant

$$g^0_{sat} = \frac{\hbar\Omega_{\mathrm{R}}}{2\chi(\mathbf{r}=0)} \int \frac{d\mathbf{p}}{(2\pi)^2}\, \chi(\mathbf{p})\chi^*(\mathbf{p})\chi(\mathbf{p}), \tag{S2}$$

where $V(\mathbf{q})$ is the Fourier image of electron-hole interaction (the Rytova-Keldysh potential), $\hbar\Omega_{\mathrm{R}}$ is the Rabi splitting, $\chi(\mathbf{r})$ and $\chi(\mathbf{p})$ are the exciton wavefunction in position and momentum bases, respectively. The expressions in Eqs. (S1) and (S2) account for electrostatic (direct and exchange) interaction and for the phase-space filling for the excitonic species of the same spin, respectively, and are valid for the case of zero temperature and the so-called rigid excitons which are strictly in $1s$ state. However, while those expressions are widely used in polariton literature, recently it has been demonstrated [5,6] that strong coupling to light modifies the exciton wavefunction.

In this section, we derive the temperature dependencies of g_{ex} and g_{sat} using the previously developed approach to bosonization in polariton systems [7] which allows to account for modifications that light-matter coupling imposes on the exciton. Our starting

point is the Bethe-Salpeter equation (BSE)

$$\left(E_{\rm g}+\frac{\hbar^2\mathbf{k}^2}{2M}+\frac{\hbar^2\mathbf{p}^2}{2m}-\mu\right)\Delta(\mathbf{p},\mathbf{k})-\int\frac{d\mathbf{q}}{(2\pi)^2}\left[V(\mathbf{p}-\mathbf{q})\ +g_{\rm R}^2\,\frac{1}{E_{\rm ph}(\mathbf{k})-\mu}\right]\Delta(\mathbf{q},\mathbf{k})=0, \quad \text{(S3)}$$

where $\Delta(\mathbf{p},\mathbf{k})$ is the exciton field dependent on the exciton c.m. momentum $\mathbf{k}$ and the relative momentum $\mathbf{p}$ of the electron-hole motion, M is the exciton mass, m is the reduced mass of an electron and a hole with respective masses m_e and m_h, $E_{\rm g}$ and μ are the exciton gap and the chemical potential, $E_{\rm ph}(\mathbf{k})$ is the photon dispersion, $g_{\rm R}$ is the amplitude of light-matter coupling. Note that in Eq. (S3) the term proportional to $g_{\rm R}^2$ desribes the influence of coupling to light on the exciton field. As it was shown in a different framework in Refs.[5,6], its presence leads to a modification of the exciton wavefunction and binding energy (compared to the solution of the standard Wannier equation) and cannot be omitted. Furthermore, $g_{\rm R}$ is connected to the experimentally-observed Rabi splitting $\hbar\Omega_{\rm R}$ via the value of the exciton wavefunction at $\mathbf{r}=0$[4,5].

Assuming that 1s excitons are resonantly coupled to light at $\mathbf{k}=0$, we separate variables in the BSE (S3) as $\Delta(\mathbf{p},\mathbf{k})=\chi(\mathbf{p})C(\mathbf{k})$, where $\chi(\mathbf{p})$ is the exciton wavefunction modified by light-matter coupling. We then relate the photon energy $E_{\rm ph}(\mathbf{k}=0)$ to the experimentally observed cavity photon resonance[5], and arrive at the equation:

$$\left(\frac{\mathbf{p}^2}{2m}-E_{\rm 1s}\right)\chi(\mathbf{p})=\int\frac{d\mathbf{q}}{(2\pi)^2}\left[V(\mathbf{p}-\mathbf{q})+\frac{(\hbar\Omega_{\rm R})^2}{4\chi^2(\mathbf{r}=0)}\frac{1}{E_{\rm ph}(0)-\mu}\right]\chi(\mathbf{q}). \quad \text{(S4)}$$

Solving this equation iteratively allows to find the modified wavefunction $\chi(\mathbf{p})$ and the exciton binding energy $E_{\rm 1s}$. Then, one can use the zero-temperature expressions for the interaction and saturation constants given by Eqs. (S1) and (S2), respectively, however using the modified exciton wavefunctions.

In Fig. S8(a) we plot the dependence of the exciton interaction constant against the exciton fraction $|X|^2$, where X is the exciton Hopfiled coefficient (in our case, taken at zero wavevector) which is defined from the experimental Rabi splitting and detuning δ as

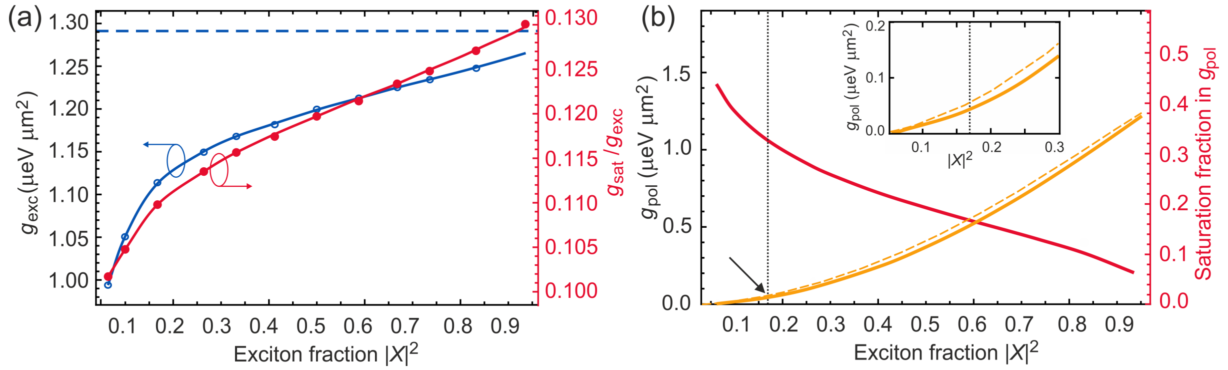

Figure S8: (a) Left axis, blue lines: exciton interaction constant g_{exc} vs. the excitonic fraction in the lower polariton given by $|X|^2$ when the light-induced modifications of the exciton wavefunction are taken into account (solid line). The dashed line shows the constant value g^0_{exc} according to (S1) in neglection of coupling to photons. Right axis, red line: the ratio g_{sat}/g_{exc} showing the increasing role of saturation with the growth of $|X|^2$. (b) The polariton nonlinearity g_{pol} for the same-spin polaritons (solid yellow lines) calculated according to Eq. (S6) vs. the excitonic fraction in the lower polariton. The dashed yellow line shows the result without light-induced modifications. The vertical dotted line and the arrow indicate the current experiment. The inset shows an enlarged view the region corresponding to large negative detunings. Right axis, red line: fraction of the blueshift coming from saturation.

$|X|^2 = (1 - \delta/\sqrt{\hbar\Omega_R^2 + \delta^2})/2$. For comparison, we also show the $g^0_{exc} \approx 1.2923$ μeV μm^2 obtained when no modification of the exciton wavefunction by light-matter coupling is considered (by the dashes line, constant value). For conditions of the our experiment, i.e. $|X|^2 = 0.167$, the values are

$$g_{exc} \approx 1.11338 \ \mu\text{eV} \ \mu\text{m}^2,$$

$$g_{sat} \approx 0.12229 \ \mu\text{eV} \ \mu\text{m}^2.$$

We note that for all detunings, the saturation constant stays one order of magnitude lower than g_{exc}. The red line in (a) shows the changing ratio g_{sat}/g_{exc} which is growing with the increase of $|X|^2$ (without light-induced modification it would be fixed at 0.136).

Finally, the theoretical lower-polariton blueshift reads (see[7] for details)

$$\Delta E_{pol} = g_{exc} n_{exc} |X|^2 + 2 g_{sat} n_{exc} |X| \sqrt{1 - |X|^2}, \tag{S5}$$

where n_{exc} is the exciton density. Comparing with the experimentally measured $\Delta E_{pol} = g_{pol} \cdot n_{pol}$ and given $n_{exc} = |X|^2 n_{pol}$ (n_{pol} is the experimentally-assessed polariton density), we conclude

$$g_{pol} = g_{exc}|X|^4 + 2g_{sat}|X|^3\sqrt{1-|X|^2}. \tag{S6}$$

For the case of circularly-polarized excitation, using the light-modified exciton interaction and saturation constants, we obtain the theoretical estimate of the same-spin polariton interaction constant from Eq. (S6)

$$g^{\text{theor}}_{pol,circ} \approx 0.04629\ \mu\text{eV}\ \mu\text{m}^2,$$

in a perfect correspondence with our experimentally assessed $g_{pol,circ} = 0.046\ \mu$eV μm^2. In Fig. S8(d) we report the behaviour of the effective polariton nonlinearity $g^{\text{theor}}_{pol,circ}$ and its contribution from the exciton saturation depending on the exciton fraction $|X|^2$. As one can see, while with the growth of $|X|^2$ the role of saturation quickly diminishes, going towards zero or positive detunings ($|X|^2 > 0.5$) leads to an increase of the polariton nonlinearity by more than an order of magnitude.

For the case of linear excitation, the usual assumption is $g_{pol,lin} = (\alpha_1 + \alpha_2)/2$, where $\alpha_1 \equiv g_{pol,circ}$ and α_2 is the interaction constant of polaritons with opposite spins. In Ref.[8] it was shown that for spin-singlet configuration the biexciton resonance may play a role, enhancing attractive interaction of opposite-spin polations. We refer to this work for calculation of the interaction constant

$$\alpha_2 = \frac{4\pi|X|^4}{M\ln|E^{\text{XX}}/E_{\text{LP}}(0)|} \approx -0.026\ \mu\text{eV}\ \mu\text{m}^2, \tag{S7}$$

where $E^{\text{XX}} = 23.3$ meV[9] is the biexciton binding energy and $E_{\text{LP}}(0)$ the energy of lower polariton at zero momentum taken with respect to the exciton resonance (-63 meV). This yields $g^{\text{theor}}_{exc,lin} = 0.01024\ \mu$eV μm^2, which is an underestimate but still in good correspondence to the measured value $g_{exc,lin} = 0.017\ \mu$eV μm^2.

As a side-note, it needs to be commented that the commonly used for zero-temperature WS_2 theoretical approximation[10,11] $g^0_{exc} = 2.07 E_\mathrm{b} \lambda_x^2$ in terms of the exciton binding energy and the 1s-exciton Bohr radius λ_x proves to be misleading. The prefactor 2.07 obtained using the hydrogenic ansatz for the exciton wavefunction $\sim \exp\{-r/\lambda_x\}$ is erroneous as it was obtained using a wrong value of the screening length of the material $\rho_0 =$ 7.5 nm in their variational procedure. Given the correct value $\rho_0 = 2\pi\alpha = 3.76$ nm, where $\alpha = 0.6$ is polarizability of WS_2 (see also[12,13]), and the Bohr radius $\lambda_x = 1.7$ nm, with the hydrogenic ansatz substituted to Eq. (S1) one gets $g^0_{exc} = 3.2$ μeV μm^2 (cf. the value 1.9 μeV μm^2 reported in[10,11]). Furthermore, substitution of the accurate (numerically simulated) shape of the exciton wavefunction in Eq. (S1) yields $g^0_{exc} = 1.2923$ μeV μm^2 which underlines as well that the hydrogenic ansatz is not to be used (it provides a good estimate for the binding energy but not for the wavefunction shape). See also Ref.[6].

References

[1] G. Moody, C. Kavir Dass, K. Hao, C.-H. Chen, L.-J. Li, A. Singh, K. Tran, G. Clark, X. Xu, G. Berghäuser, *et al.*, "Intrinsic homogeneous linewidth and broadening mechanisms of excitons in monolayer transition metal dichalcogenides," *Nature communications*, vol. 6, no. 1, p. 8315, 2015.

[2] F. Cadiz, E. Courtade, C. Robert, G. Wang, Y. Shen, H. Cai, T. Taniguchi, K. Watanabe, H. Carrere, D. Lagarde, *et al.*, "Excitonic linewidth approaching the homogeneous limit in mos 2-based van der waals heterostructures," *Physical Review X*, vol. 7, no. 2, p. 021026, 2017.

[3] F. Tassone and Y. Yamamoto, "Exciton-exciton scattering dynamics in a semicon-

ductor microcavity and stimulated scattering into polaritons," *Physical Review B*, vol. 59, p. 10830, 1999.

[4] M. Glazov, H. Ouerdane, L. Pilozzi, G. Malpuech, A. V. Kavokin, and A. D'Andrea, "Polariton-polariton scattering in microcavities: A microscopic theory," *Physical Review B*, vol. 80, p. 155306, 2009.

[5] J. Levinsen, G. Li, and M. M. Parish, "Microscopic description of exciton-polaritons in microcavities," *Physical Review Research*, vol. 1, p. 033120, 2019.

[6] D. De la Fuente Pico, J. Levinsen, E. Laird, M. M. Parish, and F. M. Marchetti, "Rydberg excitons and polaritons in monolayer transition metal dichalcogenides in a magnetic field," *Physical Review B*, vol. 111, p. 035432, 2025.

[7] A. M. Grudinina and N. S. Voronova, "Path integral approach to bosonisation and nonlinearities in exciton-polariton systems," *Physical Review B*, vol. 110, p. 115304, 2024.

[8] O. Bleu, J. Li, G.and Levinsen, and M. M. Parish, "Polariton interactions in microcavities with atomically thin semiconductor layers," *Physical Review Research*, vol. 2, p. 043185, 2020.

[9] M. Z. Mayers, T. C. Berkelbach, M. S. Hybertsen, and D. R. Reichman, "Binding energies and spatial structures of small carrier complexes in monolayer transition-metal dichalcogenides via diffusion monte carlo," *Physical Review B*, vol. 92, p. 161404, 2015.

[10] V. Shahnazaryan, I. Iorsh, I. A. Shelykh, and O. Kyriienko, "Exciton-exciton interaction in transition-metal dichalcogenide monolayers," *Physical Review B*, vol. 96, no. 11, p. 115409, 2017.

[11] F. Barachati, A. Fieramosca, S. Hafezian, J. Gu, B. Chakraborty, D. Ballarini, L. Martinu, V. Menon, D. Sanvitto, and S. Kéna-Cohen, "Interacting polariton fluids in a monolayer of tungsten disulfide," *Nature nanotechnology*, vol. 13, no. 10, pp. 906–909, 2018.

[12] T. C. Berkelbach, M. S. Hybertsen, and D. R. Reichman, "Theory of neutral and charged excitons in monolayer transition metal dichalcogenides," *Physical Review B*, vol. 88, p. 045318, 2013.

[13] M. Goryca, J. Li, A. V. Stier, T. Taniguchi, K. Watanabe, E. Courtade, S. Shree, C. Robert, B. Urbaszek, X. Marie, and S. A. Crooker, "Revealing exciton masses and dielectric properties of monolayer semiconductors with high magnetic fields," *Nature Communications*, vol. 10, p. 4172, 2019.
